# Transposable Element Interactions Shape the Ecology of the Deer Mouse Genome

**DOI:** 10.1093/molbev/msad069

**Published:** 2023-03-22

**Authors:** Landen Gozashti, Cedric Feschotte, Hopi E Hoekstra

**Affiliations:** Department of Organismic & Evolutionary Biology, Department of Molecular & Cellular Biology, Museum of Comparative Zoology and Howard Hughes Medical Institute, Harvard University, Cambridge, MA; Department of Molecular Biology & Genetics, Cornell University, Ithaca, NY; Department of Organismic & Evolutionary Biology, Department of Molecular & Cellular Biology, Museum of Comparative Zoology and Howard Hughes Medical Institute, Harvard University, Cambridge, MA

**Keywords:** mobile genetic elements, genome evolution, endogenous retrovirus, genomic conflict, *Peromyscus maniculatus*

## Abstract

The genomic landscape of transposable elements (TEs) varies dramatically across species, with some TEs demonstrating greater success in colonizing particular lineages than others. In mammals, long interspersed nuclear element (LINE) retrotransposons are typically more common than any other TE. Here, we report an unusual genomic landscape of TEs in the deer mouse, *Peromyscus maniculatus*. In contrast to other previously examined mammals, long terminal repeat elements occupy more of the deer mouse genome than LINEs (11% and 10%, respectively). This pattern reflects a combination of relatively low LINE activity and a massive invasion of lineage-specific endogenous retroviruses (ERVs). Deer mouse ERVs exhibit diverse origins spanning the retroviral phylogeny suggesting they have been host to a wide range of exogenous retroviruses. Notably, we trace the origin of one ERV lineage, which arose ∼5–18 million years ago, to a close relative of feline leukemia virus, revealing inter-ordinal horizontal transmission. Several lineage-specific ERV subfamilies have very high copy numbers, with the top five most abundant accounting for ∼2% of the genome. We also observe a massive amplification of Kruppel-associated box domain-containing zinc finger genes, which likely control ERV activity and whose expansion may have been facilitated by ectopic recombination between ERVs. Finally, we find evidence that ERVs directly impacted the evolutionary trajectory of LINEs by outcompeting them for genomic sites and frequently disrupting autonomous LINE copies. Together, our results illuminate the genomic ecology that shaped the unique deer mouse TE landscape, shedding light on the evolutionary processes that give rise to variation in mammalian genome structure.

## Introduction

Transposable elements (TEs) are parasitic genetic elements capable of mobilizing in genomes and function as important drivers of genome evolution (Orgel and Crick 1980; Kazazian 2004; Bourque et al. 2018). In mammals, for example, TEs account for at least 20% of the genome and, in some cases, have been exapted for significant functional innovations (van de Lagemaat et al. 2003; Platt et al. 2018; Senft and Macfarlan 2021). When TEs insert into new positions in the genome, they generate mutations and thus represent a significant burden on host fitness. This cost is compounded by the fact that TEs can contain gene regulatory sequences and cause structural rearrangements even after they have lost the ability to transpose (Bourque et al. 2018; Klein and O’Neill 2018). Thus, the evolutionary success of a given TE lineage is dictated by its ability to replicate faster than the host genome but limited by its cost to host fitness (Ford Doolittle and Sapienza 1980; Orgel and Crick 1980). TE lineages are in a constant coevolutionary conflict with each other as well as their host (Brookfield 2005; Venner et al. 2009). As a consequence, hosts have evolved various ways to suppress TE activity (Cosby et al. 2019). These genetic conflicts embody the “ecology of the genome” and play an important role in shaping the genomic landscape of TEs in a given species as well as its genome structure more broadly (Brookfield 2005; Venner et al. 2009).

TEs are remarkably diverse, and TE landscapes can vary dramatically across species (Wells and Feschotte 2020). TEs are classified into two broad categories based on their transposition mechanism: class I elements (retrotransposons), which mobilize through an RNA intermediate, and class II elements (DNA transposons), which do not. Most eukaryotic lineages harbor a diversity of TEs from multiple taxonomic subgroups within each of these broad classes (Bourque et al. 2018; Wells and Feschotte 2020). By contrast, some phylogenetic groups have TE landscapes that are relatively similar across species (Abrusán and Krambeck 2006; Sotero-Caio et al. 2017). One such clade is mammals (Platt et al. 2018). In most mammalian genomes, DNA transposons cannot actively mobilize and only exist as relics of anciently active elements (Platt et al. 2018). Actively mobilizing retrotransposons include long terminal repeat (LTR) retrotransposons, which are mostly endogenous retroviruses (ERVs), as well as non-LTR retrotransposons represented by long interspersed nuclear elements (LINEs) and their nonautonomous counterparts, short interspersed nuclear elements (SINEs) (Eickbush 1992; Platt et al. 2018). LINEs are nearly always the most abundant TEs, and most are represented by a single family, L1, which typically occupies hundreds of megabases of the mammalian genome (Platt et al. 2018). However, the dearth of examples of alternative TE landscapes has limited our ability to investigate the evolutionary processes driving mammalian genome structure evolution and specifically, the maintenance of LINE dominance (Furano et al. 2004; Platt et al. 2018).

The North American deer mouse, *Peromyscus maniculatus*, has become an important model for studying the genetic basis of adaptation (Bedford and Hoekstra 2015). Early studies of deer mice and closely related species used polymerase chain reaction methods to explore TE abundance and reported evidence for an unprecedented expansion of ERVs (Wichman et al. 1985; Cantrell et al. 2005). However, the landscape of TEs in the deer mouse remains unexplored on a genomic scale. Here, we report a highly distinct genomic landscape of TEs in the deer mouse genome. We find that, in contrast to nearly all examined mammalian genomes, LTR retrotransposons are more abundant in the deer mouse genome than LINEs. We investigate the evolutionary origins and implications of the deer mouse's distinct genomic landscape, revealing ecological processes that shaped its evolution.

## Results and Discussion

### Deer Mice Exhibit a Unique Landscape of TEs

**Fig. 1.**
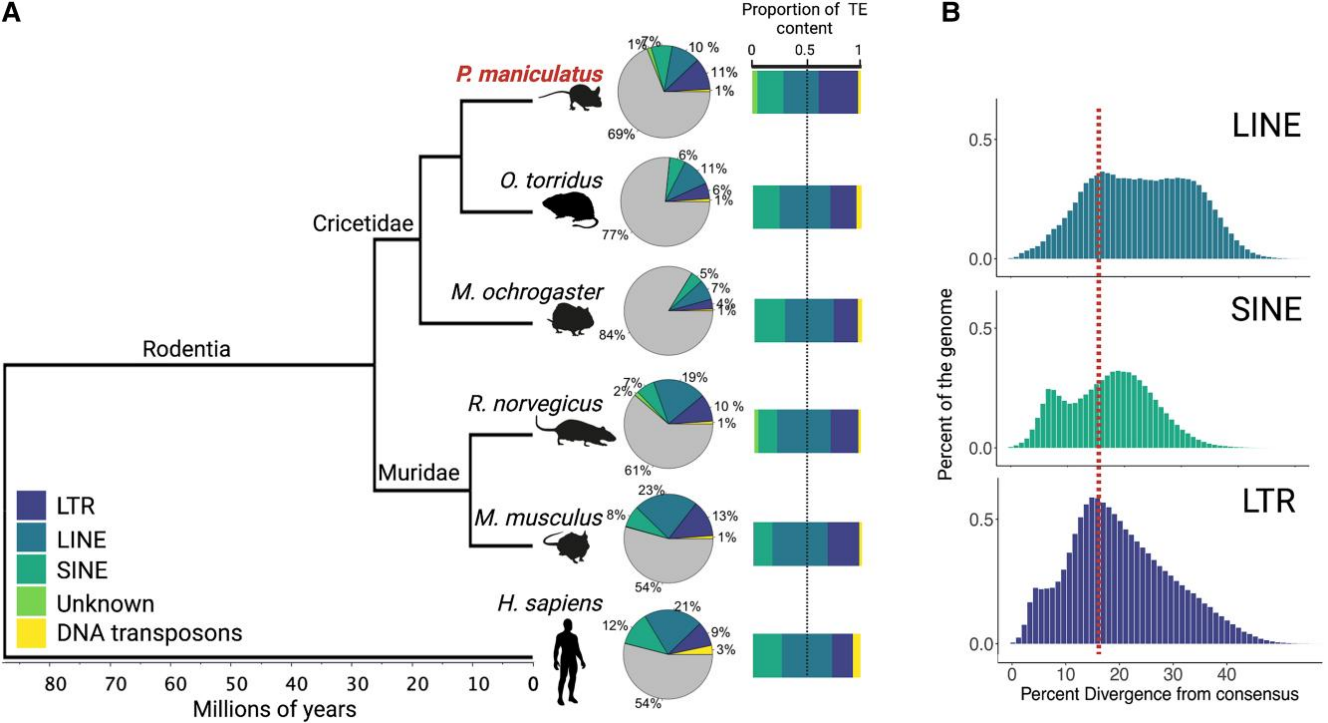
TE landscape. (*A*) Phylogeny highlighting the relationship of deer mice (*P. maniculatus*) to other mammalian species considered in this study. Branch lengths in millions of years were obtained from Timetree (Kumar et al. 2017). Pie charts show the relative percent of the genome occupied by TE subclasses for each species. Color corresponds to the percent of the genome attributed to each type of TE (see legend); gray represents the percent of the genome not occupied by TEs. Stacked bar plots show the proportion of TE content represented by each TE type. Note, in deer mouse, LTRs occupy more of the genome than LINEs. (*B*) Percent of the genome as a function of CpG corrected Kimura divergence from the consensus for each TE subfamily of LINEs, SINEs, and LTR elements. The vertical dotted line represents the start of observed LINE decline in all plots.

To evaluate the genomic landscape of TEs in the deer mouse genome, we first generated a lineage-specific TE library de novo from the deer mouse (*P. maniculatus bairdii*) genome using a combination of systematic and manual methods (see Methods). We identified 48 LINE, 28 SINE, and 118 LTR deer mouse-specific subfamilies (fig. 1*A* and supplementary table S1, Supplementary Material online). We then merged this lineage-specific TE library with all curated mammalian TEs from the Dfam database (Hubley et al. 2016) and annotated the genome using the combined library. We define lineage-specific subfamilies with respect to those observed in house mice, *Mus musculus* (strain C57BL6) (∼25 Myr diverged from the deer mouse; Kumar et al. 2017). Our annotation revealed a distinct genomic landscape of TEs in the deer mouse, relative to other mammals, in which LTR elements occupy more of the genome than LINEs (fig. 1*A*). Specifically, LTR elements occupy ∼11% of the genome, followed by LINEs (∼10%), SINEs (7%), and other TEs (<2%) (fig. 1*A* and supplementary table S2, Supplementary Material online). Notably, the 10% LINE occupancy observed for the deer mouse is much lower compared to house mouse, rat, and human. It is worth noting that the dearth of LINE content observed in the deer mouse genome is unlikely an artifact of our inability to detect lineage-specific LINEs since vertical propagation of LINEs has been accompanied by relatively little sequence changes. In total, TEs occupy ∼30% of the deer mouse genome, reflecting an increase in TE content relative to other species in the rodent Family Cricetidae, such as the grasshopper mouse (*Onychomys torridus*, 24%) and prairie vole (*Microtus ochrogaster*, 17%) (fig. 1*A*), but a reduction relative to house mouse (*M. musculus*, >40%), although differences in genome assembly and TE annotation quality may contribute to these patterns (Platt et al. 2016; Peona et al. 2021). Nonetheless, most of the difference in TE content between the deer mouse and house mouse can be attributed to decreased LINE content in the deer mouse, whereas most of the difference in TE content among cricetid species can be attributed to LTR elements.

Based on these observations, we hypothesized that the distinct TE landscape of deer mice is the result of a combination of reduced accumulation of lineage-specific LINE1s (L1s) and a proliferation of lineage-specific LTR elements. To investigate this possibility, we first compared genomic representation as a function of within-subfamily divergence (as a proxy for subfamily age) across LINEs, SINEs, and LTR elements (fig. 1*B*). Consistent with our hypothesis, we observe reduced representation of LINEs with lower divergence from the consensus, suggesting decreased LINE accumulation in the deer mouse lineage on more recent timescales (fig. 1*B*). However, despite this decline in the accumulation of LINEs, we still find multiple candidate L1s with intact protein machinery, suggesting that LINEs are still active, consistent with previous reports of LINE activity in deer mice (supplementary table S3, Supplementary Material online; Casavant et al. 1996). We also observed evidence for lineage-specific SINE activity (fig. 1*B*). Since SINEs parasitize LINE machinery for mobilization, evidence of recently active SINEs suggests that recently active LINEs still exist in the genome. In addition, we find a lineage-specific proliferation of LTR elements (fig. 1*B*): LTR elements are significantly overrepresented among the youngest TEs in the genome (<1% divergence from the consensus; two-sided Fisher's exact test, *P* < 0.00001). Furthermore, the observed decline of LINE gains in the genome coincides with the peak of LTR gains in the genome (fig. 1*B*). Together, these results suggest that both reduced LINE gain and lineage-specific LTR proliferation have contributed to the deer mouse's unique TE landscape, and that the two may be associated.

### DNA Loss Fails to Explain Reduced LINE Content

**Fig. 2.**
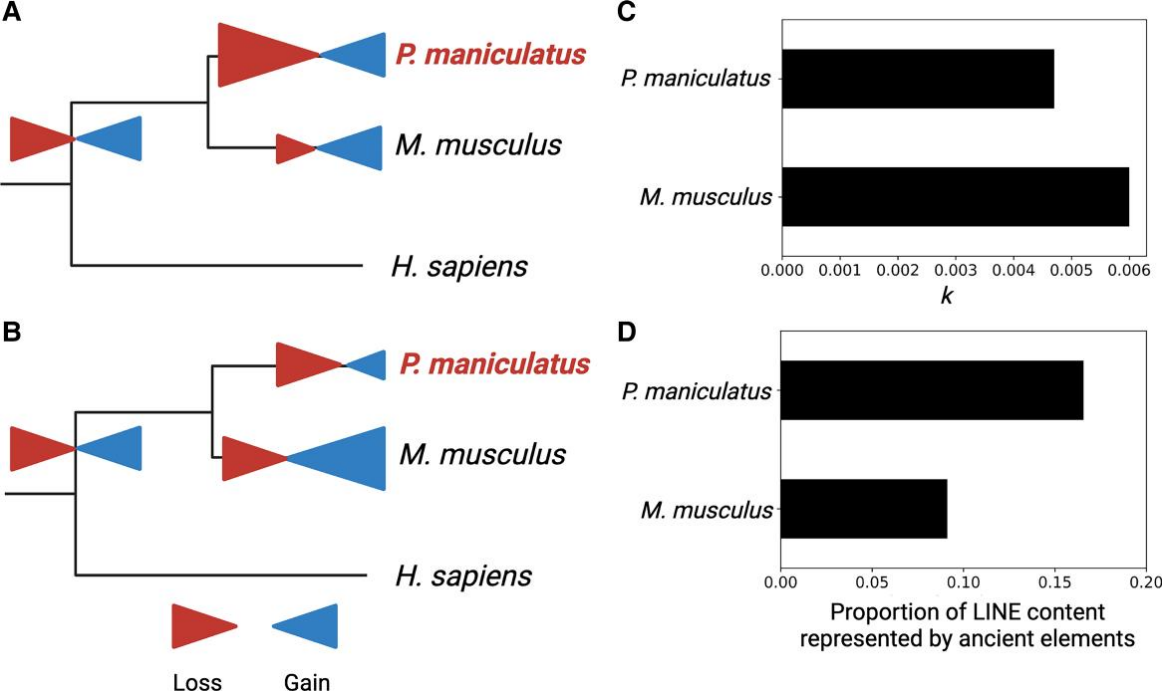
Non-mutually exclusive evolutionary scenarios that may have shaped the TE landscape in the deer mouse. (*A*) Higher rates of lineage-specific loss (larger red arrows) could have resulted in the reduced LINE content observed in deer mouse (*P. maniculatus*) relative to house mouse (*M. musculus*) and/or (*B*) higher rates of lineage-specific gain (larger blue arrows) in the house mouse relative to the deer mouse. (*C*) *k* coefficients of DNA loss suggest lower rates of loss in deer mouse relative to house mouse. (*D*) Ancient elements account for a greater proportion of total LINE content in deer mouse relative to house mouse.

In addition to gain, TE loss can be an important driver of genomic TE content (Kapusta et al. 2017). Although we find evidence for a decline of LINE gain, the low LINE content in the deer mouse genome, relative to house mouse, could also have resulted from higher rates of ancestral DNA loss in the deer mouse (fig. 2*A* and *B*). To investigate this possibility, we calculated the DNA loss coefficient *k* (following Lindblad-Toh et al. 2005), using the formula *E* = *Ae* − *kt*, where *E* is the amount of extant ancestral DNA in the species considered, *A* is the ancestral assembly size, and *t* is time. Larger values of *k* suggest higher rates of lineage-specific DNA loss. We calculated a *k* coefficient of ∼0.0047 for the deer mouse, a value similar to, and in fact slightly lower than the *k* value estimated for the house mouse (∼0.006; Kapusta et al. 2017). These data suggest that the reduced LINE content observed in the deer mouse genome cannot be explained by generally higher rates of DNA loss in deer mice (fig. 2*C*).

While the results above indicate that the genome-wide rate of DNA loss cannot explain the low LINE content of the deer mouse genome, it is still possible that LINEs are lost at a higher rate than other types of TEs. To investigate this possibility, we compared the proportions of DNA attributed to ancient mammalian LINEs present in the common ancestor of the deer mouse and house mouse as well as lineage-specific elements. If the relative absence of LINEs in the deer mouse is due to higher rates of loss of these types of elements, we expect to find a decreased amount of DNA attributed to ancient LINEs in the deer mouse relative to house mouse. To the contrary, we find that although LINEs contribute twice as much content to the house mouse as to the deer mouse (∼575Mb vs. ∼250Mb), ancient LINEs are significantly underrepresented in the house mouse genome (∼16% of total LINEs in deer mouse vs. ∼9% of total LINEs in house mouse; two-sided Fisher's exact test, *P* = 0.006; fig. 2*D*). These results suggest that LINE DNA is lost at a slower rate in the deer mouse lineage than in the house mouse lineage, consistent with our *k* calculations above. Together these data suggest that the low LINE content observed in the deer mouse cannot be attributed to high rates of LINE DNA loss, and instead, is most likely the result of relatively low rates of LINE activity in the deer mouse lineage.

### ERVK Elements Amplified Predominantly as Nonautonomous Subfamilies

**Fig. 3.**
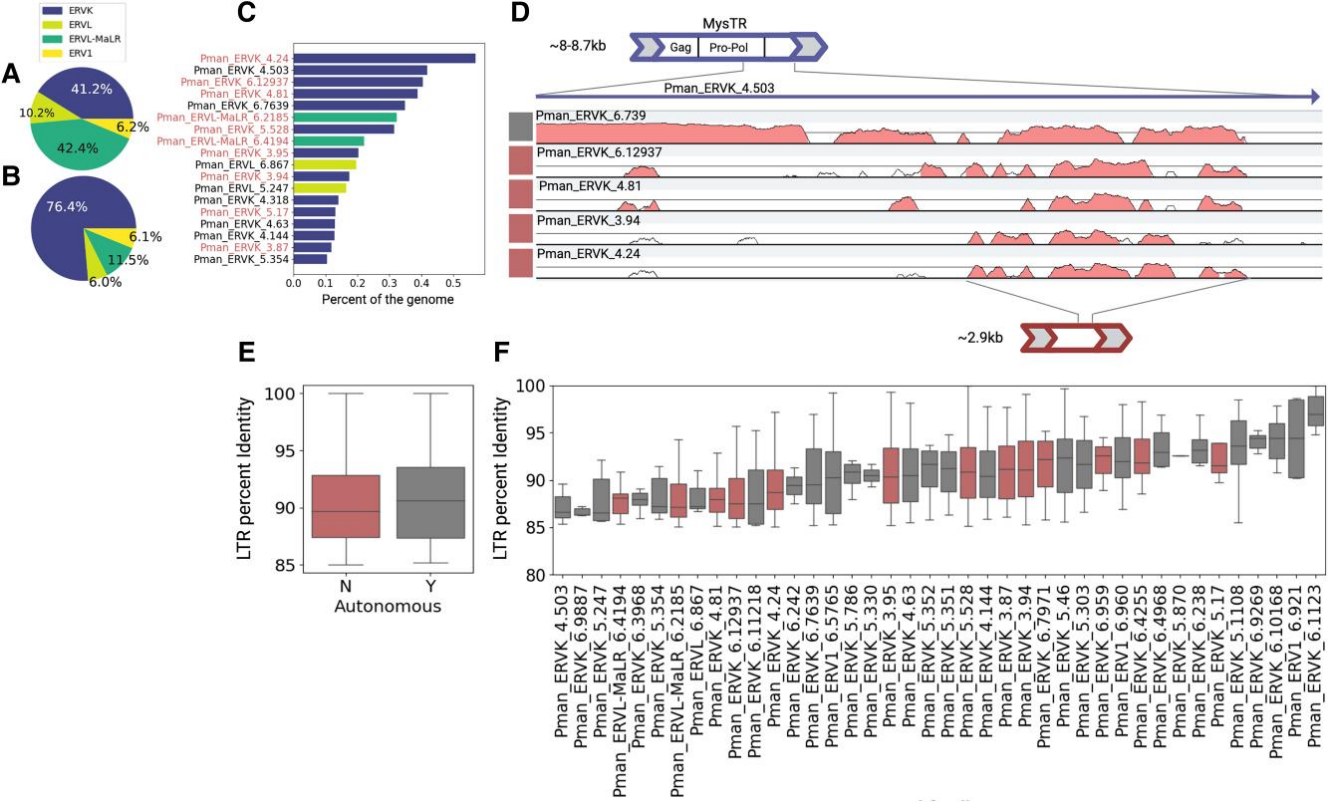
Relative contribution of different broad ERV classes to ERV content in the deer mouse genome across (*A*) all ERVs and (*B*) lineage-specific ERVs. (*C*) Respective genomic occupancy across the 18 most common lineage-specific ERV subfamilies. Red font denotes nonautonomous subfamilies. (*D*) VISTA plot showing regions of homology between and relative position of autonomous mysTR subfamilies (gray) and nonautonomous subfamilies (red). (*E*) Comparison of LTR percent identity aggregated across all lineage-specific autonomous and nonautonomous elements. (*F*) Comparison of LTR percent identity across all ERV subfamilies that display at least one candidate full-length copy showing that the youngest subfamilies are autonomous (gray).

Most mammalian LTR retrotransposons are represented by ERVs, which are derived from germline infiltrations of exogenous retroviruses (Mager and Stoye 2015). ERVs are divided into three broad classes depending on their retroviral origins: ERV1 (Gammaretroviridae), ERVK (Betaretroviridae), and ERVL (Spumaretroviridae), with a subgroup of nonautonomous ERVLs called MaLRs (Hubley et al. 2016; Gifford et al. 2018). In the deer mouse, we find a pattern in which ERVK and ERVL/MaLR elements together account for over 80% of genomic ERV content, consistent with previous reports in other rodents (Hubley et al. 2016; Platt et al. 2018; fig. 3*A*). Lineage-specific ERVs as a whole represent over half of genomic ERV content (∼57%), suggesting that the deer mouse has experienced a substantial ERV expansion. When we compare the proportion of lineage-specific ERV content to the proportion of shared ERV content represented by ERVKs, we find that ERVKs account for a disproportionately large part of lineage-specific ERV content (two-sided Fisher's exact test, *P* < 0.00001), representing over 75% of observed lineage-specific ERV sequence in the genome (fig. 3*B* and supplementary table S1, Supplementary Material online). Thus, ERVK activity has been particularly pronounced in the deer mouse lineage.

In well-annotated mammalian genomes, among full-length proviral elements (with two LTRs), ERVKs are typically represented by autonomous elements that encode their own machinery for mobilization (Mager and Stoye 2015). After manually inspecting deer mouse*-*specific ERV subfamilies, and annotating *gag*, *pro*, *pol*, and *env* genes as well as predicting protein domains required for autonomous transposition, we find that the most abundant ERVK families do not possess any internal open reading frames (ORFs) predicted to encode proteins with conserved domains, suggesting that they are largely composed of nonautonomous elements. Many ERVs contain assembly gaps that interrupt or truncate their internal sequences (507 of 1,119 candidate full-length ERVs), making it challenging to reconstruct full-length elements and assess the presence or absence of coding machinery. In light of this caveat, we required that a putatively nonautonomous ERV subfamily display at least five full-length copies with no gaps for it to be classified as nonautonomous, regardless of its consensus sequence length or content. Even with this conservative filter, we find that the most abundant deer mouse-specific ERV subfamilies are nonautonomous ERVK-like elements lacking any obvious coding capacity (fig. 3*C* and supplementary table S4, Supplementary Material online). Pman_ERV2_4.24, for example, is the most abundant ERV in the genome, accounting for ∼5% of total ERV content. Furthermore, for the subset of ERVKs in which we could confidently reconstruct full-length copies and assess their coding capacity, nonautonomous elements occupy more of the genome than autonomous ones (supplementary table S4, Supplementary Material online). Overall, our results suggest that autonomous ERVKs and their nonautonomous counterparts have had a significant impact on the deer mouse's genome structure.

Nonautonomous TEs parasitize autonomous elements for mobilization. Studies on the nonautonomous ERVK subfamily, ETn, in house mouse showed that ETn exhibits regions of sequence similarity to fully coding MusD elements, suggesting that ETn likely arose from the ancestors of Mus-D (Mager and Freeman 2000) and now hijacks MusD machinery for mobilization via trans-complementation (Ribet et al. 2004). Given the high copy numbers of nonautonomous ERVKs in the deer mouse genome, we sought to identify related autonomous elements that may have been their progenitors and/or facilitated their mobilization. In addition to several prolific nonautonomous subfamilies, we identified three autonomous ERVK subfamilies, Pman_ERVK_4.503, Pman_ERVK_6.7639, and Pman_ERVK_5.247, that together occupy ∼1% of the genome (fig. 3*C*). These subfamilies show sequence similarity to mysTR (∼89%, ∼96%, and ∼90% identity to mysTR pro-pol sequence, respectively), an ERV family previously identified in *Peromyscus* (Cantrell et al. 2005), suggesting a shared origin from mysTR. Together, these mysTR-related subfamilies represent the most abundant autonomous ERVs in the genome.

Previous studies failed to identify full-length mysTR copies with intact *gag* and *pro-pol* genes required for mobilization, raising questions about its overall origin and ability to mobilize, although these studies lacked the genomic resources to analyze mysTR sequences comprehensively (Cantrell et al. 2005; Erickson et al. 2011). Our genome-wide analysis reveals multiple copies of mysTR*-*related ERVs, which display apparently intact ORFs with homology to *gag* and *pro-pol* genes and contain all the protein domains expected to be encoded by autonomous elements, suggesting that these ERVs are indeed autonomous and may still be capable of mobilizing (supplementary table S4, Supplementary Material online). We also find several nonautonomous subfamilies related to mysTR (fig. 3*D* and supplementary table S4, Supplementary Material online; Wichman et al. 1985; Lee et al. 1996). The most conserved region of nucleotide sequence homology between mysTR-related subfamilies is just downstream of the *pro-pol* gene and upstream of the 3′ LTR (fig. 3*D*). Interestingly, most candidate nonautonomous and autonomous mysTR-related subfamilies do not display strong homology outside of this region, suggesting that nonautonomous subfamilies may have evolved through a recombination event in an autonomous element, which replaced the original internal sequence of the autonomous element with a nonhomologous sequence (Mager and Freeman 2000). Maintenance of sequence similarity in this region is also consistent with functional constraint due to a possible role in ERVK mobilization, although its function remains unknown.

Since ERV LTRs are identical upon insertion, LTR sequence identity can provide an estimate for how recently an ERV inserted. To investigate the evolutionary dynamics of deer mouse ERVs, we compared the distributions of LTR identity across lineage-specific ERV subfamilies. We find that nonautonomous ERVs overall exhibit similar ages to autonomous ERVs (fig. 3*E*). However, when we compare nonautonomous and autonomous subfamilies independently, we observe evidence for waves of autonomous element activity followed by waves of nonautonomous element activity, with the most recently active subfamilies being autonomous (fig. 3*F*). These observations are consistent with the hypothesis that nonautonomous subfamilies evolved from autonomous subfamilies.

### Diverse Origins of ERVs

ERVs arise in a species when an exogenous retrovirus infects the germline. Thus, new families of ERVs evolve de novo through horizontal introduction more frequently than other autonomous mammalian TEs such as LINEs, which primarily evolve through the diversification of vertically inherited elements (Mager and Stoye 2015). To investigate the origins of ERVs in the deer mouse, we focused on full-length ERVs across all identified subfamilies with flanking LTRs, *pol* genes, and reverse transcriptase (RT) domains (>450 bp), which we used for classification and phylogenetic analysis. We initially identified 148 candidate full-length ERVs with *pol* genes and evidence of an RT domain. However, many ERVs contained ambiguous sites or gaps that interrupted or truncated the RT domain, leaving only 52 ERVs that met our conservative requirements (supplementary tables S5 and S6, Supplementary Material online). Thus, we note that our estimate of ERV diversity is likely an underestimate.

**Fig. 4.**
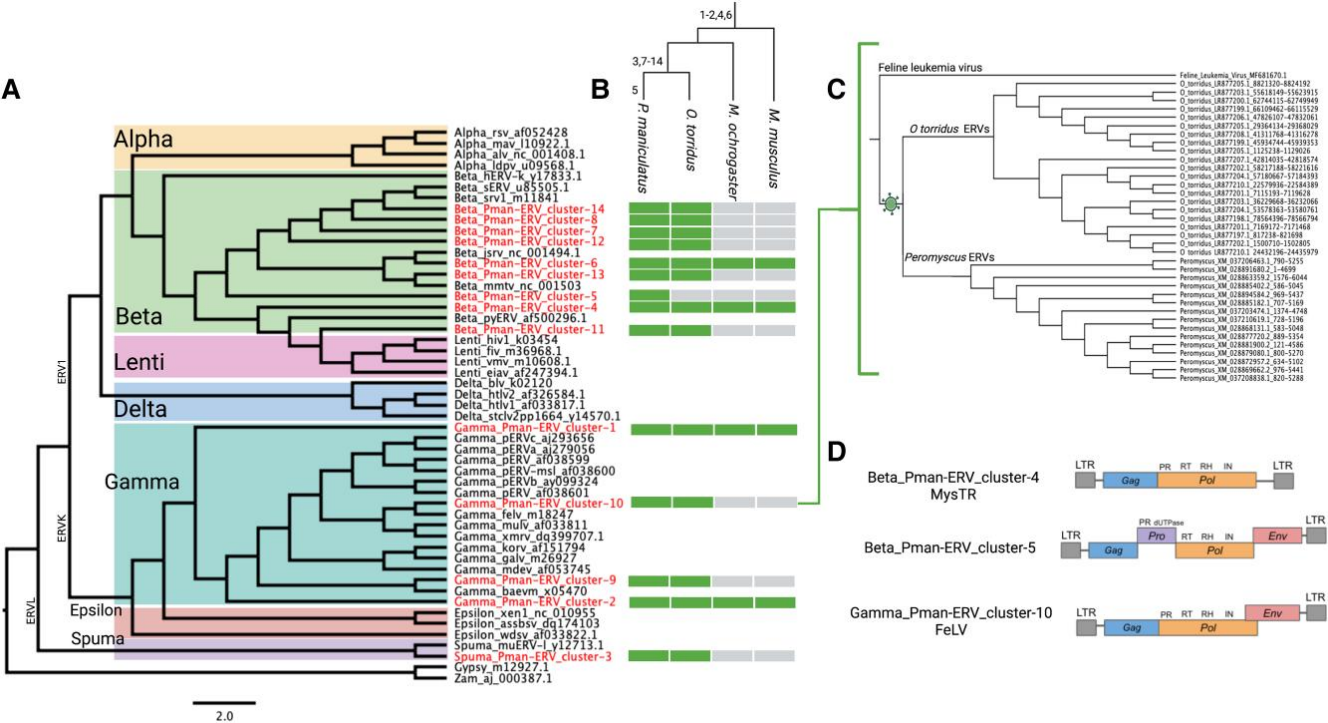
Origins of ERVs in the deer mouse genome. (*A*) Cladogram displaying a maximum likelihood tree constructed with *RT* domains of deer mouse ERVs and publicly available endogenous and exogenous retroviruses for context. Internal nodes across monophyletic retroviral clades are all strongly supported (>95% bootstrap support). Deer mouse ERVs form 14 distinct clusters spanning broad retroviral diversity (highlighted in red). (*B*) Cladogram showing the approximate time of origin of deer mouse (*P. maniculatus*) ERVs based on the presence or absence in three other species at increasing phylogenetic distances. ERV cluster numbers are shown on the branch corresponding to their approximate origin. For each species, green boxes represent presence and gray boxes represent absence for each respective ERV cluster found in deer mouse. (*C*) Neighbor-joining tree showing the phylogenetic relationship between Pman-ERV_cluster-10 copies in deer mouse, related ERVs in grasshopper mouse (*O. torridus*), and FeLV. (*D*) Structure of ERVs found in deer mouse with ORFs (colored boxes); important protein domains are annotated. Overlapping boxes represent overlapping ORFs.

We initially used a hidden Markov model approach (Finn et al. 2011) to classify ERVs based on their RT domains (supplementary table S6, Supplementary Material online). Using this approach, we find that of the 52 deer mouse ERVs with full-length RT domains, 11 are derived from gammaretroviruses (ERV1), 39 from betaretroviruses (ERVK), and 2 from spumaretroviruses (ERVL) (supplementary table S6, Supplementary Material online). Phylogenetic analysis of RT domains from these ERVs and other known retroviruses supports these initial classifications and shows that deer mouse ERVs form 14 distinct clusters representing at least 14 independent endogenization events spanning retroviral diversity (fig. 4*A*). Most of these are derived from diverse betaretroviruses (9 of the 14), consistent with observations in other rodents (Baillie et al. 2004; Cui et al. 2015). Additionally, four ERV clusters show evidence of gammaretroviral origin, and one ERV cluster shows evidence of spumaretroviral origin (fig. 4*A*).

To determine the age of ERVs, we conducted searches for deer mouse ERVs in grasshopper mouse, prairie vole, and house mouse. We find that most (9 of the 14) deer mouse ERVs arose before the divergence of the deer mouse and its close relative, the grasshopper mouse (∼5–13 million years ago [MYA]) (León-Paniagua et al. 2007; Leite et al. 2014), but after the divergence of their ancestor and the lineage of the prairie vole (∼18 MYA) (Abramson et al. 2009; Kumar et al. 2017), and are thus lineage-specific relative to house mouse (fig. 4*B*). Additionally, one ERV (Beta_Pman-ERV_cluster-5) was introduced even more recently, after the divergence between the deer mouse and grasshopper mouse (fig. 4*B*). Intra-element LTR identity for ERVs in each respective cluster generally concurs with the timing estimate of their successive endogenization (supplementary table S6, Supplementary Material online). Given the relatively recent origins of several deer mouse ERVs (since the divergence between *Peromyscus* and *Microtus* ∼18 MYA), we reasoned that it may be possible to trace more precisely their origins by searching the databases for their closest exogenous retrovirus relatives. This search revealed one potential case of a recent endogenization of an exogenous Feline Leukemia Virus (FeLV), or a closely related virus, in the ancestor of the deer mouse and grasshopper mouse within the last ∼5–18 million years (fig. 4*C*; Abramson et al. 2009; Kumar et al. 2017).

### Some Deer Mouse ERVs may Still be Infectious

Although ERVs only require *gag* and *pol* genes to mobilize in the germline via retrotransposition, ERVs with intact *env* genes can also replicate via reinfection (Belshaw et al. 2004). Given the relatively recent evolution of several ERVs in the deer mouse, we inspected all intact ERVs as well as ERV subfamily consensus sequences for intact *env* genes. We find no evidence for *env* genes in mysTR-related subfamilies, consistent with previous studies on mysTR (Cantrell et al. 2005; fig. 4*D* and supplementary table S4, Supplementary Material online). However, we find putatively full-length *env* genes in multiple other ERV clusters, suggesting that some deer mouse ERVs may still be capable of infection (supplementary table S4, Supplementary Material online). One of these is the previously mentioned FeLV-related Gamma_Pman-ERV_cluster-10. The observation of a putatively intact *env* in these ERVs is consistent with previous studies showing that leukemia viruses remain infectious in other species (Hoover and Mullins 1991; Polat et al. 2017; fig. 4*D*). We also observe evidence of an intact *env* gene for ERVs within the Beta_Pman-ERV_cluster-5. Beta_Pman-ERV_cluster-5 ERVs are absent in grasshopper mice and thus represent some of the most recent ERVs to infiltrate the deer mouse germline (fig. 4*B* and *D*). Interestingly, *env* genes from this family show ∼60% sequence similarity to the *env* encoded by intracisternal A-type particle (IAP) elements in house mice, which are also capable of intercellular transmission (Ribet et al. 2008), suggesting a possible origin from a similar exogenous retrovirus (supplementary tables S4 and S6, Supplementary Material online). Together these data suggest that several ERVs derived from exogenous retroviruses recently and some may still be infectious.

### Negative Selection Shapes TE Distributions in the Deer Mouse Genome

**Fig. 5.**
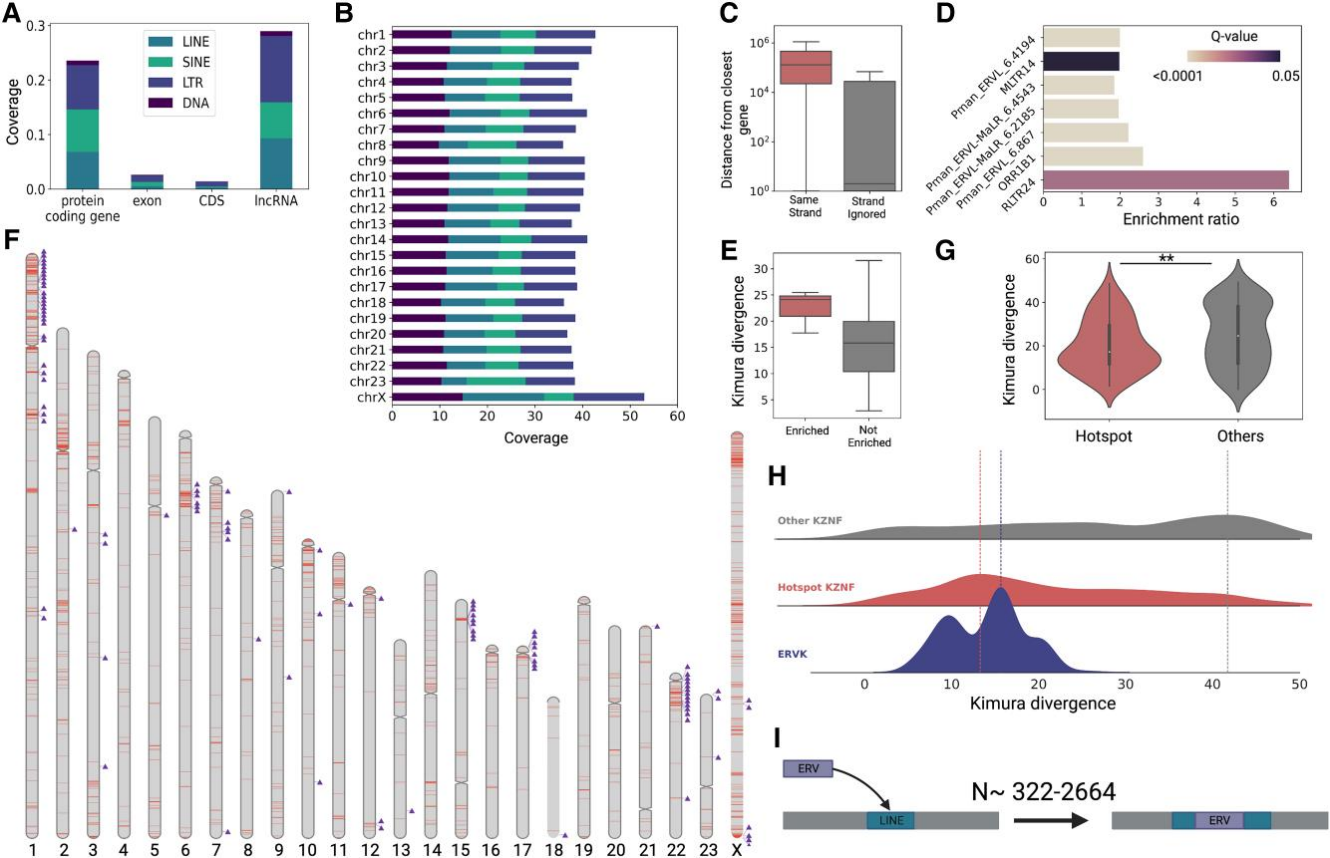
Genomic distribution of TEs in the deer mouse genome. (*A*) Respective coverage, defined as the proportion of nucleotides attributed to TEs for a given feature, for different TE subclasses across genomic features. CDS = protein coding sequence; lncRNA = long noncoding RNA. (*B*) Respective coverage for TEs across chromosomes. (*C*) Box plots showing the distribution of ERV distances from the closest gene on the same strand (red) versus when strand is ignored (gray). (*D*) Enrichment ratio (number observed/expected) and Bonferroni-corrected Fisher's exact test *P*-values (*Q*-values) for ERV subfamilies enriched within the 5 kb region upstream of genes in the same orientation. (*E*) Within-subfamily CpG corrected Kimura divergence for ERV subfamilies enriched within the 5 kb region upstream of genes in the same orientation (red) compared to all other ERV subfamilies (gray). (*F*) Genomic distribution of ERV hotspots (red) across chromosomes. Lineage-specific KZNF genes are indicated (purple triangles) and are enriched in ERV hotspots. (*G*) KZNFs in ERV hotspots (red) show lower Kimura divergence than other KZNFs (gray), suggesting that they are younger. (*H*) Kernel density estimates for the distribution of Kimura divergences for KZNFs outside ERV hotspots (gray), KZNFs in ERV hotspots (red), and ERVKs (blue). Vertical dotted lines show the peak value for each distribution. (*I*) Cartoon displaying an ERV insertion interrupting a formerly intact LINE. *N* represents the range of observed candidate instances of ERV-mediated LINE interruption.

Most TE insertions are deleterious or neutral, and the genomic distribution of TEs is shaped in large part by selection against deleterious insertions. In the deer mouse genome, TEs account for nearly 25% of nucleotides in protein coding genes and 30% in long noncoding RNAs (lncRNAs) nucleotides but are relatively absent from coding exons (permutation test, *P* < 0.001), suggesting strong purifying selection on new insertions in coding exons (fig. 5*A*). These patterns are consistent with observations in other mammals (Nellåker et al. 2012; Kapusta et al. 2013; Platt et al. 2018). Comparison of TE occupancies across chromosomes reveals that ERVs and LINEs are prevalent on the X chromosome (occupying ∼15% and ∼17 of the X chromosome, respectively, compared to an average of ∼12% and ∼10% for other chromosomes; fig. 5*B*). This pattern is not observed for SINEs and likely reflects the more frequent removal of longer TEs such as LINEs and ERVs on autosomes by recombination or purifying selection against new insertions (Kent et al. 2017; Dechaud et al. 2019). ERV insertions around protein coding genes are also usually deleterious since ERVs contain complex internal regulatory elements that can disrupt gene expression. Consistent with this, ERVs are generally distant from genes and significantly more distant from genes in the same orientation (Mann Whitney U, *P* < 0.0001; fig. 5*C*). It is worth noting that this bias is most pronounced for mysTR-related ERV subfamilies, suggesting that these ERVs are highly deleterious, perhaps containing regulatory sequences particularly prone to disrupt gene expression when inserted in the same orientation as surrounding genes.

### Some ERV Subfamilies Have Possible Regulatory Function

While most ERV subfamilies show patterns suggesting deleterious effects affecting the expression of neighboring genes, others display patterns consistent with possible regulatory function. Indeed, ERV LTRs may be co-opted for important regulatory functions over evolutionary time (Chuong et al. 2017). We find a small subset of ERV subfamilies to be enriched in the 5-kb region upstream of gene transcription start sites, suggesting that these ERVs minimally affect neighboring gene expression or that they may contribute to host gene regulation as either promoters or enhancers (fig. 5*D*). These ERVs also display significantly higher within-subfamily divergence relative to other lineage-specific deer mouse ERVs (Mann Whitney U, *P* = 0.0048), suggesting that they primarily represent older, inactive subfamilies (fig. 5*E*). Additionally, some subfamilies, including MT2B1 and ORR1B1, represent lineages of ancestrally shared elements that have been co-opted for regulatory functions in other mammalians (Franke et al. 2017). Given the frequent and recurrent lineage-specific ERV co-option events observed across mammals (Feschotte 2008; Sakashita et al. 2020; Fueyo et al. 2022), these subfamilies represent promising candidates for co-option events in the deer mouse lineage.

### ERVs Accumulate in “Hotspots” Enriched for Kruppel-Associated Box-Zinc Finger Genes

The distribution of ERVs in the genome is largely biased towards specific regions, or “hotspots”, which are enriched in Kruppel-associated box (KRAB) domain-containing zinc finger genes (KZNFs). We define “hotspots” as regions of the genome in the top 95th percentile of ERV density, where ERV density is the proportion of nucleotides attributed to ERVs in a given 100-kb genomic window (fig. 5*F*). Lineage-specific ERVKs constitute over 70% of ERVs in hotspots, suggesting that these genomic associations are likely lineage-specific. Furthermore, neighboring ERVs are significantly more divergent in ERV hotspots than in other regions of the genome (Mann Whitney U, *P* = 3.317e−06), suggesting that hotspots arose primarily through independent insertions rather than segmental duplication (SD) of existing insertions. ERV hotspots are largely devoid of genes, and we observe a strong negative correlation between gene density and ERV density overall (generalized linear model, *P* < 0.0001). However, we do observe some genes in ERV hotspots. We performed a gene ontology (GO) enrichment analysis for genes in ERV hotspots and found significant enrichment for one biological process term: “regulation of transcription, DNA-templated” (two-sided Fisher's exact test, *Q* < 0.00001). Scrutiny of genes overlapping ERV hotspots that match this GO term reveals that ∼85% (100/118) are deer mouse-specific KZNFs (fig. 5*C*). We define deer mouse-specific KZNFs based on refseq's annotation of genes that do not have orthologs in other species (O’Leary et al. 2016). We find that deer mouse-specific KZNFs specifically are enriched in ERV hotspots, with ∼32% (100/312) of KZNFs occurring in ERV hotspots, despite the fact that ERV hotspots only represent <5% of the genome (two-sided Fisher's exact test, *P* < 0.00001).

### Coevolution of ERVs and KZNFs

It has become increasingly clear that the primary function of KZNFs is to suppress retroelement activity (Thomas and Schneider 2011; Yang et al. 2017; Cosby et al. 2019). KZNF gene clusters evolve rapidly through a birth-death model under positive selection and often expand in response to the lineage-specific activity of retroelements, including ERVs (Emerson and Thomas 2009; Najafabadi et al. 2015; Wolf et al. 2020). The colocalization of KZNF genes and ERVs in genomic space is intriguing and has been observed previously in the house mouse (Kauzlaric et al. 2017). Although this observation could simply be explained by relaxed selection on nonessential KZNF genes, two alternative, non-mutually exclusive hypotheses could explain the observed colocalization between KZNFs and ERVs: (1) KZNFs use neighboring ERVs as regulatory sequences to respond to the global derepression of ERVs (Pontis et al. 2019; Ito et al. 2020) or (2) ERVs contribute to KZNF gene family evolution by facilitating rapid gene duplication and deletion (i.e., turnover) in these regions. Indeed, ERVs are known to facilitate structural rearrangements via ectopic recombination, and ERV-rich regions of the genome can be highly plastic (Hughes and Coffin 2001; Doxiadis et al. 2008; Jern and Coffin 2008; Hermetz et al. 2012). Interestingly, lineage-specific KZNF duplicates in ERV hotspots exhibit significantly lower divergence compared to other KZNFs, suggesting that genes in ERV hotspots duplicated relatively recently (Mann Whitney U, *P* = 0.0043; fig. 5*G*). This observation supports the idea that KZNFs overlapping with ERV hotspots duplicate more often, although the evolutionary processes driving this pattern remain unclear.

Next, we examine whether lineage-specific KZNF gene family expansion located in ERV hotspots coincides with lineage-specific ERV activity. To do so, we compared the age (sequence divergence) distribution of KZNF gene duplicates located within ERV hotspots to that of KZNFs residing outside ERV hotspots and that of ERVK subfamilies, as measured by intra-subfamily copy divergence (fig. 5*H*). The distribution of duplicate divergence for KZNFs in ERV hotspots suggests that the largest KZNF expansion occurred just before or around the same time as the peak of ERVK amplification (fig. 5*H*). Indeed, the median percent divergence for lineage-specific KZNF gene duplicates in ERV hotspots is ∼17.2%, while the within-subfamily divergence for the top three most abundant ERVs in the deer mouse genome is ∼17.4%. This pattern is consistent with a KZNF expansion driven by the amplification of highly active lineage-specific ERVKs. In contrast, the distribution of duplicate divergence for KZNFs not overlapping ERV hotspots shows no obvious relationship to lineage-specific ERVK activity (fig. 5*H*), further suggesting that the observed colocalization between ERVKs and KZNFs may be causally associated. Furthermore, some KZNF gene clusters display much larger expansions than others: for example, a cluster on chromosome 1 contains >90 genes, representing about one-third of lineage-specific KZNFs in the deer mouse genome (fig. 5*F*). This observation suggests that KZNFs in this chromosome 1 cluster may play an important role in suppressing ERVKs. We observe another example on chromosome 22, which displays a cluster of 48 lineage-specific KZNF genes. Since members of the same KZNF clusters often bind to related ERV families, the massive invasion of closely related ERVs predicts expansions of closely related KZNFs (Wolf et al. 2020). Together, these results suggest that KZNFs in the deer mouse underwent a large expansion in response to lineage-specific ERV activity.

### Lineage-Specific ERV Insertions Interrupt LINE Sequences

In addition to evaluating ERV distributions with respect to genes, we also assessed ERV distributions with respect to other TEs. We were specifically interested in how the observed ERV invasion in the deer mouse might directly impact pre-existing LINEs. Specifically, we sought to examine whether ERVs could have directly impacted L1 activity by inserting into and interrupting transposition-competent L1. L1 families typically only have from a hundred to a few thousand “master genes” that are transposition-competent in mammalian genomes (Deininger et al. 1992; Brouha et al. 2003; Zemojtel et al. 2007; Platt et al. 2018). Furthermore, the L1 retrotransposition mechanism is fairly inefficient, and the vast majority of new L1 insertions are defective and incapable of mobilizing thereafter (Fanning 1983; Grimaldi et al. 1984). Thus, disruption of many master genes could have a considerable impact on the evolutionary trajectory of L1s in a species.

To explore the direct impacts of ERV insertions on L1s, we searched for ERV insertions directly flanked by L1 sequences from the same L1 subfamily. We then filtered for cases in which flanking L1 sequences conjoined at the correct coordinates with respect to the subfamily consensus, forming a full-length L1. We also initially filtered for L1s that did not contain any additional TE insertions. These results revealed 322 prospective lineage-specific ERV insertions that interrupt full-length L1 (supplementary table S7, Supplementary Material online). However, this number is likely a large underestimate, since it does not include fragmented L1s that, for example, have accumulated multiple indels. If we include fragmented L1s as well, we find 2,664 prospective ERV insertions interrupting LINEs, 900 of which are attributed to the two most abundant ERVK related subfamilies (Pman_ERV2_4.503 and Pman_ERV2_4.24; supplementary table S8, Supplementary Material online). Interestingly, within-subfamily percent divergences for these subfamilies (16.05% and 15.86%) suggest that they invaded just before the decline of L1 gain (see fig. 1*B*, ∼15%). We speculate that this association is no coincidence, and that ERV insertions within potentially active L1s were a significant driver in reducing L1 activity in the deer mouse lineage. Punctuated L1 interruptions on these scales (322 - > 2,500 L1 interruptions) would eliminate most functional L1s in many mammalian species, and even on much smaller scales, could have a catastrophic effect on the evolutionary trajectory of L1s in a genome, especially given the poor success rates of the L1 retrotransposition mechanism in producing new fully functional L1 copies (Kazazian and Moran 1998; Szak et al. 2002).

We also explored quantitative patterns of ERV content in LINEs more broadly. To do so, we tested for an enrichment of ERVs inserted within L1s across all ERV subfamilies. Interestingly, we find that several lineage-specific ERVK subfamilies show significant enrichment within L1s relative to random expectation (Bonferroni-corrected permutation test, *α* = 0.01, *N* = 1000; supplementary table S9, Supplementary Material online). For example, Pman_ERVK_4.63 elements interrupt L1s more than 46 times more than expected by chance (Bonferroni-corrected permutation test, *Q* < 0.001). The observed enrichment for lineage-specific ERVK subfamilies, representing 29 of the 36 enriched subfamilies, and absence of enrichment for other (older) ERV subfamilies, is consistent with our hypothesis that lineage-specific ERVK expansion directly impacted L1 viability (supplementary table S9, Supplementary Material online). Together, these results suggest an intriguing model whereby ERVs and LINEs compete for genomic sites and that ERVKs may have directly impacted the evolutionary trajectory of LINEs in the deer mouse lineage.

### An “Ecology of the Genome” Model for the Evolution of the Deer Mouse Genome

In the same way that species compete for space and resources, TEs compete with each other for sites in the genome as well as metabolic resources (Brookfield 2005). TEs can occupy specific niches, which can allow them to coexist with limited competition, but TEs that occupy similar niches are more likely to compete and thereby drive one or another to extinction (Brookfield 2005; Venner et al. 2009). Furthermore, the relative success of a given TE also depends on host suppression mechanisms and their targets. For example, differential host targeting between two TE families in direct competition could limit the success of one family that would, in the absence of host defense mechanisms, be more fit than the other (Venner et al. 2009). Also, because TEs could threaten to kill their host in the absence of host-mediated suppression, it can be advantageous (for both the host and TEs) that host defenses evolve to suppress TE activity (Venner et al. 2009).

**Fig. 6.**
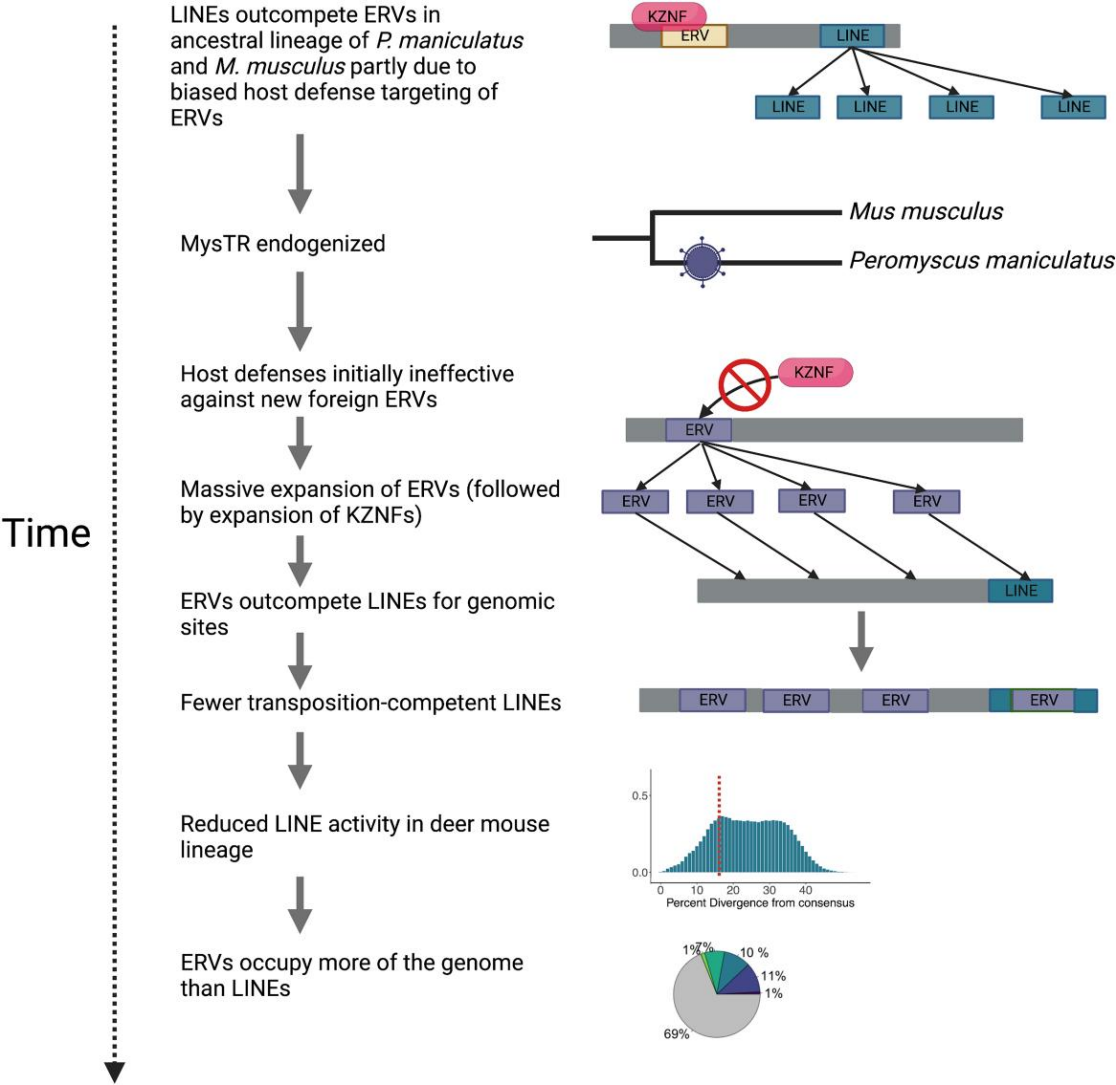
Model for deer mouse genome evolution.

Our model for the evolution of the distinctive TE landscape of the deer mouse supports the notion of “genomic ecology” (Brookfield 2005). We postulate that the introduction of mysTR-related ERVs caused a shift in the deer mouse TE landscape through the following processes (fig. 6): first, mysTR ERVs evaded host defenses upon germline infiltration, which allowed them to expand to large numbers. This hypothesis is supported by the observation that mysTR ERVs are highly divergent from other known retroviruses as well as the remarkable expansion of deer mouse-specific KZNFs following peak ERV activity (Cantrell et al. 2005). In mammals, ERVs are the primary targets of KZNF suppression, whereas LINEs and SINEs are less frequently targeted, probably because ERV insertions are more regulatorily potent and therefore more deleterious (Wolf et al. 2015; Zhou et al. 2020). These host defenses keep ERVs in check, despite evidence that LINEs and ERVs compete for similar sites in the genome. First, many ERVs and LINEs both preferentially integrate into AT rich regions (Medstrand et al. 2002; Babushok et al. 2006; Nellåker et al. 2012; Campos-Sánchez et al. 2016). Thus, ERVs and LINEs often inhabit similar regions of the genome and frequently insert within each other (Campos-Sánchez et al. 2016). Second, ERV insertions in LINEs (or vice versa) are likely invisible to selection and exhibit a higher rate of fixation relative to deleterious insertions (Campos-Sánchez et al. 2016). Under these circumstances, in the absence of host defense mechanisms, we expect the primary driver of ERV or LINE success in the genome to be relative rates of gain of transposition-competent copies. Thus, we postulate that the massive expansion of mysTR ERVs nearly drove LINEs to extinction in the deer mouse genome. Since this initial ERV invasion, we postulate that expansions of host KZNF repertoires helped stabilize ERV activity in the genome and were likely aided by the proliferation of nonautonomous ERV derivatives. These suppression mechanisms likely enabled more sustainable ERV activity by limiting the rate of ERV expansion and reducing fitness cost.

More generally, we propose that this model may explain the loss of LINE activity in other mammals. A subclade of sigmodontine rodents for example (∼13–18 MYA diverged from the deer mouse; Abramson et al. 2009; Leite et al. 2014; Kumar et al. 2017; Gonçalves et al. 2020) represents one of the few mammalian lineages to have experienced LINE extinction (Yang et al. 2019). Consistent with our model, previous studies suggest that LINE extinction in this group followed an invasion of mysTR-related ERVs on a similar or possibly larger scale to that observed for the deer mouse (Cantrell et al. 2005; Erickson et al. 2011). At present, the lack of genome assemblies for sigmodontine rodents makes it challenging to study TEs in these species. However, a recent study examining TE content in mammals notes that cricetid rodents exhibit the highest rates of recent LTR retrotransposon accumulation among mammals (Osmanski et al. 2022). Overall, we hypothesize that for an ERV invasion to have a similar effect on LINE activity in another mammal, the causal ERV must arise from a divergent retrovirus (unfamiliar to host suppression machinery), show similar integration preference to that of LINEs, and rapidly evolve nonautonomous derivatives. Future studies in other species, which show unique patterns of mammalian genome composition, will shed further light on evolutionary conflicts that drive mammalian genome evolution.

### Concluding Remarks

Although TE landscapes differ drastically across species, most mammalian genomes are similarly dominated by LINE (and SINE) non-LTR retrotransposons. The deer mouse, *P. maniculatus*, represents one of the few exceptions to this pattern: LTR elements occupy more of the genome than LINEs. We find that the distinct genomic landscape of TEs in the deer mouse reflects a massive expansion of ERVs as well as a dearth of LINE activity, and that the two phenomena are likely associated. Our results show that a broad diversity of ERVs invaded the deer mouse genome and that the infiltration of one ERV family in particular, mysTR, played a prominent role in establishing its unique TE landscape. Furthermore, we note that reported ERV copy numbers and diversity are likely a vast underestimate since the current deer mouse genome assembly was generated with short reads (Peona et al. 2021).

Based on these findings, we postulate that the propensity for a mammalian genome to undergo a shift in TE content and/or experience LINE extinction is directly related to its susceptibility to invasion by divergent TEs—in this case, ERVs. Furthermore, the accumulation of ERVs in specific genomic hotspots raises additional questions about how TE-dense regions can affect mammalian genome evolution. Indeed, we would expect such regions to experience structural rearrangements more often than other regions of the genome. Previous studies in the deer mouse have identified many large inversions (>1 Mb in length), which are polymorphic, even within populations (Hager et al. 2022; Harringmeyer and Hoekstra 2022). Could these ERV hotspots have played a role in facilitating deer mouse inversions? We also observe enrichment of KZNF gene families which evolve rapidly via duplication within ERV hotspots. Is the colocalization of KZNFs and ERVs advantageous for the host due to the increased propensity for KZNF gene family expansion? We show that KZNFs in ERV hotspots are indeed younger than other KZNFs, providing some support for the coevolution of these genomic features. However, within-population studies are critical to further elucidate this coevolutionary relationship. Together, our results have broad implications and open up a range of opportunities to investigate the evolutionary processes that give rise to the evolution of mammalian genome structure.

## Methods

### Obtaining Relevant Genomic Data

We downloaded publicly available TE annotations for human, *Homo sapiens* (GCF_000001405.40; genome contig N50 = 57,879,411; contig L50 = 18); house mouse, *M. musculus* (GCF_000001635.27; genome contig N50 = 59,462,871; contig L50 = 15); Norway rat, *Rattus norvegicus* (GCF_015227675.2; genome contig N50 = 29,198,295; contig L50 = 27); and prairie vole, *M. ochrogaster* (GCF_000317375.1; genome contig N50 = 21,250; contig L50 = 29,205) from RepeatMasker (http://www.repeatmasker.org/genomicDatasets/RMGenomicDatasets.html) and for grasshopper mouse, *O. torridus* from NCBI (GCF_903995425.1; genome contig N50 = 2,276,141; contig L50 = 308). We used the deer mouse, *P. maniculatus*, genome assembly available through NCBI (refseq GCF_003704035.1; contig N50 = 30,111; contig L50 = 23,323) for all genomic analyses. Retroviral sequences for ERV phylogenetic analysis were downloaded from NCBI. Genbank accession numbers and for these sequences are shown in fig. 4*A*.

### TE Discovery and Annotation

We used a combination of systematic and manual techniques to identify and annotate TEs in the deer mouse genome. We started by using an approach similar to the EarlGrey pipeline (github.com/TobyBaril/EarlGrey/; Baril and Hayward 2022). We first identified known rodent TEs in the deer mouse genome using RepeatMasker (version 4.1.2, https://www.repeatmasker.org/) with a curated set of rodent TEs from the DFAM database (Hubley et al. 2016) and the flags -*nolow*, *-norna*, and -*s*. Next, we constructed a de novo repeat library using RepeatModeler2 (version 2.0.1), with RECON (version 1.08) and RepeatScout (version 1.0.5) (Bao and Eddy 2002; Price et al. 2005; Flynn et al. 2020). Maximum-length consensus sequences were generated for putative de novo TEs identified by RepeatModeler using an automated version of the “Basic Local Alignment Search Tool (BLAST), Extract, Extend” process through EarlGray (Platt et al. 2016). Briefly, EarlGray first performs a BLASTn search to obtain the top hits for each TE subfamily (Camacho et al. 2009). Then, it aligns the 1,000 base pairs of flanking retrieved sequences using multiple alignment using fast fourier transform (MAFFT; version 7.453; Katoh and Standley 2013). Following this, alignments are trimmed using trimAl (version 1.4) with the options (-*gt* 0.6 *-cons* 60; Capella-Gutiérrez et al. 2009). Finally, consensus sequences are updated using European molecular biology open software suite cons (-*plurality* 3; Rice et al. 2000). This process is then repeated five times. Following this, we performed blastx (Camacho et al. 2009) searches against all known deer mouse proteins with parameters (*-max_target_seqs* 25 *-culling_limit* 2 *-evalue* 10e-10) and filtered all TEs with unknown classifications that shared homology with proteins.

Following the automated processes described above, alignments for TE families were individually inspected using AliView (Larsson 2014) and poorly represented positions were manually trimmed as recommended by Storer et al. (2021). Families were also manually realigned using extract_align.py (Platt et al. 2016) and MAFFT (version 7.453; Katoh and Standley 2013) and then reexamined. Manually curated TE families were then re-clustered using cd-hit-est (Fu et al. 2012) and families were merged based on the 80-80-80 rule criterion (Wicker et al. 2007). We also used TE-Aid (https://github.com/clemgoub/TE-Aid) to identify TE-associated ORFs and sequence features such as LTRs when classifying TEs. We combined our final de novo TE library with the Rodent DFAM TE library (Hubley et al. 2016) and annotated TEs in the deer mouse genome using RepeatMasker. To identify full-length LTR elements, we used LTR_FINDER (Xu and Wang 2007) and LTRharvest (Ellinghaus et al. 2008) through EDTA_raw with the flag *-type ltr* (version 2.0.0; Ou et al. 2019), which also report LTR divergence for each element. TE annotations were defragmented and refined using RepeatCraft with the flag -*loose* (Wong and Simakov 2019), and overlapping annotations were resolved in favor of the longer element using MGKit (version 0.4.1) filter-gff (Rubino and Creevey 2014).

### Identifying Functional Machinery for Putatively Autonomous TEs

To identify the protein machinery of potentially autonomous LINE and LTR elements, we extracted all LINE elements longer than 2700 bp and LTR elements longer than 5000 bp from the deer mouse genome. Then, we also used TE-Aid (https://github.com/clemgoub/TE-Aid) to identify ORFs in each retrieved LTR and LINE element with homology to known TE genes. We used hmmer (Finn et al. 2011) and relevant hmms available from GyDB (Llorens et al. 2011) and PFAM (Mistry et al. 2021) to identify retroviral protein domains as well as NCBI's conserved domain search tool (Marchler-Bauer et al. 2015; Marchler-Bauer et al. 2017).

### Calculating *k* Coefficients

We calculated the DNA loss coefficient *k* (Lindblad-Toh et al. 2005), using the formula *E* = *Ae* − *kt*, where *E* is the amount of extant ancestral DNA in the species considered, *A* is the ancestral assembly size, and *t* is the time. We calculated *E* for each species by subtracting the amount of genomic DNA attributed lineage-specific TEs from the amount of DNA attributed to ancient shared mammalian TEs (retrieved from Kapusta et al. 2017). We used 2.8 Gb for *A* and 100 million years for *t* as in Kapusta et al. (2017).

### Identifying Nonautonomous ERV-Like Elements

Since the deer mouse genome was produced primarily with short reads, most ERVs have internal gaps or strings of low quality or ambiguous nucleotides. Thus, to decipher nonautonomous ERV-like elements from autonomous ERVs, we used a strict criterion. For a given ERV subfamily to be considered nonautonomous, we required at least five full-length copies which lack identifiable ORFs as well as ambiguous nucleotides. We performed global pairwise alignments between nonautonomous and autonomous ERVK consensus sequences using the global alignment software AVID with default parameters (Bray et al. 2003). We visualized alignments using VISTA (Frazer et al. 2004).

### ERV Classification and Phylogenetic Analysis

We used two complementary approaches to classify deer mouse ERVs. First, we examined *e*-value statistics in the output from our GyDB hmm scans to discern which viral RT domain hmm best fit each ERV. In addition, we also used a phylogenetic approach. We annotated ERVs with their viral origin as predicted by our hmm scans. Next, we downloaded several endogenous and exogenous retroviruses from NCBI (accessions shown in fig. 4*A*), extracted their RT domains, and annotated them with their respective viral clade. Then, we filtered sequences with large strings of ambiguous characters, performed a multiple sequence alignment of RT genes using MAFFT (Katoh and Standley 2013), and generated a maximum likelihood-based phylogeny using IQ-TREE (Minh et al. 2020) with a GTR + G model (general time reversible model with unequal rates and unequal base frequencies and discrete gamma rate heterogeneity; Yang 1994). We constructed a consensus tree across 1,000 replicates using IQ-TREE's *-bb* flag (Yang 1994). All internal nodes separating monophyletic ERV clades were strongly supported (>95% bootstrap support). We analyzed and edited the resulting phylogeny using ete3 (version 3.1.2) (Huerta-Cepas et al. 2016), collapsing clusters of deer mouse ERVs into representative nodes. We visualized the phylogenetic tree using the interactive tree of life (Letunic and Bork 2021) and FigTree (version 1.4.4; https://github.com/rambaut/figtree). To search for homology between deer mouse ERVs and MysTR, we used blastn (Camacho et al. 2009) to align deer mouse ERV consensus sequences to a previously isolated MysTR pol-pro sequence (Genbank DQ139737.1; Cantrell et al. 2005).

### Searching for Deer Mouse ERVs in Other Species

To search for deer mouse ERVs in the house mouse, prairie vole, and grasshopper mouse genomes, we performed local BLASTn (Camacho et al. 2009) queries for each full-length deer mouse ERV to each respective genome. We ran BLASTn (Camacho et al. 2009) with the flag *-outfmt 6* and required a minimum alignment length of 400 bp and minimum percent identity of 75 to limit possible erroneous hits. As a proof of concept, we also made sure our results were consistent with expectations based on LTR divergences. For example, we would not expect an ERV with highly divergent LTRs (a signature of a more ancient insertion) to be specific to the deer mouse. We also performed broader BLASTn queries against NCBI's nucleotide database. Queries for Gamma_Pman-ERV_cluster-10 sequences yielded high-confidence hits in deer mouse species, the grasshopper mouse, and a FeLV reference genome (Genbank AB060732.3). BLAST queries of FeLV (AB060732.3) back to the non-redundant nucleotide database also showed best hits to the deer mouse and grasshopper mouse genomes when other FeLV genomes were excluded. A neighbor-joining phylogeny constructed from deer mouse Gamma_Pman-ERV_cluster-10 sequences, homologous ERVs in the grasshopper mouse genome, and FeLV (AB060732.3) suggest a scenario in which Gamma_Pman-ERV_cluster-10 originated in the common ancestor the deer mouse and grasshopper mouse from FeLV or another closely related exogenous virus between 5 and 18 MYA.

### TE Distribution Analysis

We used bedtools intersect (Quinlan and Hall 2010) to find overlaps between TE annotations and gene feature annotations. We used bedtools closest (Quinlan and Hall 2010) with the parameter -*s* to identify TE distances from the nearest gene on the same strand and again with default parameters to ignore strand. All functional enrichment tests were performed using goatools (Klopfenstein et al. 2018). We also tested for enrichment or depletion of TEs 5 kb upstream of genes in the same orientation. Specifically, for each TE subfamily, we randomized all TE locations on each chromosome and compared the number of TEs within 5 kb of genes upstream in the same orientation with the observed value. We performed two-sided Fisher's exact tests comparing the number of observed and expected elements within these regions to obtain *P*-values. Fisher's exact *P*-values and permutation test *P*-values were adjusted using the Bonferroni method to obtain *Q*-values. This revealed eight subfamilies that displayed enrichment for the 5 kb regions upstream of genes in the same orientation (supplementary table S10, Supplementary Material online). However, enrichment of SDs in these regions could also cause a similar pattern. To test this alternative, we used SEDEF (version 1.1) (Numanagic et al. 2018) with default parameters to identify SDs in the deer mouse genome. Then, for each enriched ERV subfamily, we intersected SD coordinates with the 5 kb regions upstream of genes harboring at least one element in the same orientation. We then compared SD coverage in these regions for each ERV subfamily with expectations from randomization for 1,000 permutations. This revealed that genes containing RMER15 copies within 5 kb upstream in the same orientation also displayed enrichment for SDs, suggesting that SDs could alternatively explain RMER15 (supplementary table S10, Supplementary Material online). Thus, we did not include RMER15 in figure 5*D*. We used bedtools coverage (Quinlan and Hall 2010) to calculate ERV and gene density along 100 kb windows in the genome. ERV hotspots were defined as windows which exhibit ERV densities within the top 95th percentile. ERV hotspots could arise through two possible non-mutually exclusive mechanisms: independent insertion of ERVs in specific genomic regions and SD of pre-existing ERV insertions. One expectation of the latter is that neighboring ERVs of the same subfamily would exhibit more similar divergences from the consensus in ERV hotspots (if they arose from a duplication of the one original insertion) than in other regions of the genome. To assess this possibility, we compared delta divergence from the consensus between neighboring ERVs (|neighbor_1_div—neighbor_2_div|) in ERV hotspots to other regions of the genome and report a significant trend in the opposite direction (Mann Whitney U, *P* = 3.317e−06), suggesting that SD is not the primary contributing mechanism to ERV hotspot formation. Figure 5*F* was produced using RIdeogram (Hao et al. 2020).

### KZNF Gene Family Analysis

We defined deer mouse-specific zinc finger (ZF) genes as genes which do not have recognizable orthologs as annotated by NCBI. We employed hmmscan (Finn et al. 2011) using KRAB hmms downloaded from PFAM (Mistry et al. 2021) to identify KRAB domain-containing ZFs (KZNFs). Then, we performed a multiple sequence alignment of all KZNFs using Clustal omega (Sievers et al. 2011) with the parameters *-use-kimura* and *-full* in order to simultaneously produce a pairwise Kimura divergence matrix across all genes. We constructed a subsequent phylogeny using IQ-TREE (Minh et al. 2020) with a general time reversible model. To test for phylogenetic clustering of KZNF that overlapped ERV hotspots, we used phyloclust through RRphylo R package (Castiglione et al. 2018) with 100 simulations. Since KZNF genes evolve via a birth-death process, we define duplicate genes as genes that exhibit the lowest divergence among all pairwise comparisons.

### ERV-Mediated LINE Interruption

To identify candidate LINEs interrupted by ERVs, we searched for LINE fragments which would be full length (>5000 bp) if connected but exhibit an ERV sequence which splits them with respect to their subfamily consensus (supplementary table S7, Supplementary Material online). This yielded 322 candidate ERV-mediated LINE interruptions, 121 of which represented lineage-specific LINEs. In this first analysis, we excluded LINEs which showed more than two fragments. If we include those as well, we find 2,664 candidate ERV-mediated LINE interruptions. We employed a permutation test to quantitatively assess biased representation of ERVs in LINEs. We did this separately for each ERV subfamily. To do this, we compared the observed number of ERV insertions inside LINEs (ERV sequences flanked on both sides by LINE sequences from the same subfamily) to expectations by randomization 1,000 times. We calculated the proportion of iterations that ERVs interrupted LINEs more than expected to obtain a *P*-value for each ERV subfamily. Then we performed a Bonferroni correction to obtain *Q*-values (supplementary table S8, Supplementary Material online).

## Supplementary Material

msad069_Supplementary_Data

## Data Availability

No new data were generated in support of this research. TE models were deposited in the DFAM database.
