# Supplementary material for: Transposable Element Interactions Shape the Ecology of the Deer Mouse Genome
Source: Mol Biol Evol. 2023 Mar 22;40(4):msad069. doi: 10.1093/molbev/msad069 (PMC10089650; doi:10.1093/molbev/msad069)
Supplement: msad069_Supplementary_Data [file msad069_supplementary_data.zip › Gozashti_MBE_Supplement.pdf]

Supplementary Tables

S1 Table: Lineage specific transposable element subfamilies. Absolute and wellchar length of the genome occupied by each lineage-specific TE subfamily as well as average within subfamily CpG corrected Kimura divergence.

S2 Table: RepeatMasker summary table. Summary of RepeatMasker output, showing the number of elements and genomic occupancy across broad TE taxonomic groups.

S3 Table: Candidate autonomous LINE copies. LINE copies >5000 bp in length with potentially intact *pol* genes required for transposition. Columns **A-D** show the genomic position and strand for each candidate autonomous LINE. Columns **E-H** note the position of candidate *pol* genes with respect to each LINE copy as well as homologous *pol* genes as identified by BLAST. Column **I** shows additional notes for each candidate autonomous LINE copy after manual inspection.

S4 Table: Summary of ERV subfamilies. Summarizing information for each ERV subfamily with at least one candidate copy showing an internal sequence flanked by LTRs on each side. Columns **K-R** show best hit known ERV *gag*, *pro*, *pol*, and *env* genes for orfs as well as orf positions with respect to each subfamily consensus. Comma separated homologous gene names in a single cell denote a fragmented orf (either due to a stop codon or gap) in which different fragments yield different best hits.

S5 Table: Filtering ERVs for phylogenetic analysis.

S6 Table: Candidate ERVs with intact RTs for phylogenetic analysis. *TE*: ERV genomic position and strand. *Cluster:* ERV phylogenetic cluster. *Related subfamilies:* ERV subfamilies which display homology to candidate intact ERVs for phylogenetic analysis. Columns **D-J** show positions of protein domains identified using HMMs with respect to each ERV. Columns **K-S** positions of orfs for *gag*, *pro-pol*, and *env* with respect to each ERV as well as best hit homologous genes from known ERVs.

S7 Table: Candidate ERV-mediated L1 interruptions with fragmented L1s excluded. *Start1/Stop1* and *Start2/Stop2* denote the start and stop for each L1 fragment separated by an ERV insertion. Columns **F-J** denote the ERV subfamily responsible for each candidate L1 interruption as well as the position of the specific insertion in the genome.

S8 Table: Candidate ERV-mediated L1 interruptions with fragmented L1s included. Columns are the same as in Supplementary Table 7. *Start1/Stop1* and *Start2/Stop2* denote the start and stop for each L1 fragment separated by an ERV insertion. Columns **F-J** denote the ERV subfamily responsible for each candidate L1 interruption as well as the position of the specific insertion in the genome.

S9 Table: Permutation test results for ERV preference in L1s. Permutation tests were performed independently for each subfamily. Q-values represent Bonferroni-corrected P-values. Enrichment ratio is defined as the number of candidate observed ERV insertions in L1s over the expected number ERV insertions in L1s.

S10 Table: ERV subfamilies enriched in 5 kb regions upstream of genes in the same orientation. For each ERV subfamily we performed Fisher's exact tests for enrichment in 5 kb regions upstream of genes in the same orientation with respect to a random distribution. We then performed a Bonferroni correction on these P-values to obtain reported Q-values. Only subfamilies with significant Q-values (<0.05) are shown. Enrichment ratio is defined as (number of observed)/(number of expected) elements observed. We also report enrichment ratios and permutation test results for overlaps with segmental duplications, which could alternatively explain the observed pattern of enrichment upstream of genes in the same orientation.
